# A Color Hierarchy for Automatic Target Selection

**DOI:** 10.1371/journal.pone.0009338

**Published:** 2010-02-24

**Authors:** Illia Tchernikov, Mazyar Fallah

**Affiliations:** 1 Centre for Vision Research, York University, Toronto, Ontario, Canada; 2 School of Kinesiology and Health Science, York University, Toronto, Ontario, Canada; 3 Canadian Action and Perception Network, Toronto, Ontario, Canada; University College London, United Kingdom

## Abstract

Visual processing of color starts at the cones in the retina and continues through ventral stream visual areas, called the parvocellular pathway. Motion processing also starts in the retina but continues through dorsal stream visual areas, called the magnocellular system. Color and motion processing are functionally and anatomically discrete. Previously, motion processing areas MT and MST have been shown to have no color selectivity to a moving stimulus; the neurons were colorblind whenever color was presented along with motion. This occurs when the stimuli are luminance-defined versus the background and is considered achromatic motion processing. Is motion processing independent of color processing? We find that motion processing is intrinsically modulated by color. Color modulated smooth pursuit eye movements produced upon saccading to an aperture containing a surface of coherently moving dots upon a black background. Furthermore, when two surfaces that differed in color were present, one surface was automatically selected based upon a color hierarchy. The strength of that selection depended upon the distance between the two colors in color space. A quantifiable color hierarchy for automatic target selection has wide-ranging implications from sports to advertising to human-computer interfaces.

## Introduction

In sports competitions the outcome of the match is thought to rely on the abilities of the players not the color of the uniforms. When referees monitor the match, do they pay attention to each team equally? In life red is often used for stop or danger, whereas green is used for go or safe. We may pay more attention to red than green because of their meanings. Does one football team draw more penalties because their red uniforms are more salient to the referees? Is a baseball player sliding in to the plate more likely to be called safe rather than out, if the color of his uniform captures the umpire's attention? If this color bias occurs automatically, then it could impact our perception and judgments without us being aware of it.

Visual processing of color is well understood from cones in the retina through ventral stream color selectivity via the parvocellular pathway [1]. Color processing has been distinct from motion processing [1]–[3] which is mediated by the magnocellular system [4]. However, it is possible to perceive motion that is only defined by color [5], [6]. In chromatic motion processing, the border between 2 isoluminant colors moves, e.g. a red-green grating. Behavioral and neuronal responses to this motion are attenuated[7]–[10] compared to most of the motion we see around us that is defined by achromatic, or luminance-based, motion processing [11]. Neurons in motion processing areas MT and MST have been shown to have no color selectivity. The neurons did not respond differently to a moving bar as its color was changed [12]. Chromatic properties have been shown to provide no real benefit to luminance defined motion processing [10]. Yet color differences can be used to filter out distractors from targets in a motion discrimination task, both behaviorally in humans [13] and in the behavior and MT neuronal responses in primates [14]. In these latter studies, color filtering was part of the task demand for top-down selection of the target and suppression of the distractors.

We tested whether color could automatically bias selection, as has been shown previously with contrast [15]. Our results show that achromatic motion processing is intrinsically modulated by color. We measured smooth pursuit eye movements automatically produced upon saccading to an aperture containing coherently moving dots. Color modulated smooth pursuit both when there was only a single surface of dots or two surfaces that differed in color. Furthermore, selection between colors followed a color hierarchy and the strength of the motion modulation was related to the distance between the two colors in color space.

## Results

**Figure 1:**
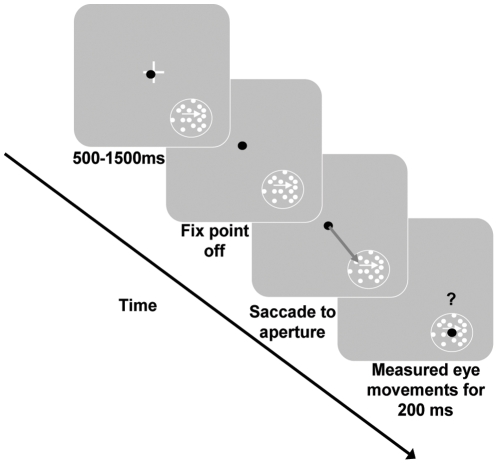
Experimental paradigm. Subjects fixated (eye position – black dot) on the white fixation cross (0.5°) at which point a 5° aperture appeared at 7° eccentricity in either the lower right or lower left quadrants. After a random period of time (500–1500 ms) the cross disappeared which was the signal to saccade to the aperture. The surface(s) in the aperture continued to move. Smooth eye movements were measured from 50–200 ms post-saccade. In Experiment 1, the aperture contained a single surface moving at either left or right (shown) and was 1 of 4 possible isoluminant colors (red, blue, green, or yellow). In Experiment 2, the aperture contained two superimposed surfaces moving in opposite directions. The surfaces were comprised of 1 of 6 possible color combinations: red-green, red-blue, red-yellow, green-blue, green-yellow and blue-yellow.

As shown in Figure 1, when a subject fixated on a central cross, a static aperture containing a single colored moving surface appeared. When the cross disappeared, subjects had to saccade to the aperture. It is important to note that making an accurate saccade was the subject's only task demand. As a result of selecting the surface as the saccade target, subjects automatically pursued the moving surface. This likely occurs due to the overlap in neural circuitry controlling saccadic and pursuit eye movements [16]. Smooth pursuit was analyzed from 50–200 ms post-saccade.

**Figure 2:**
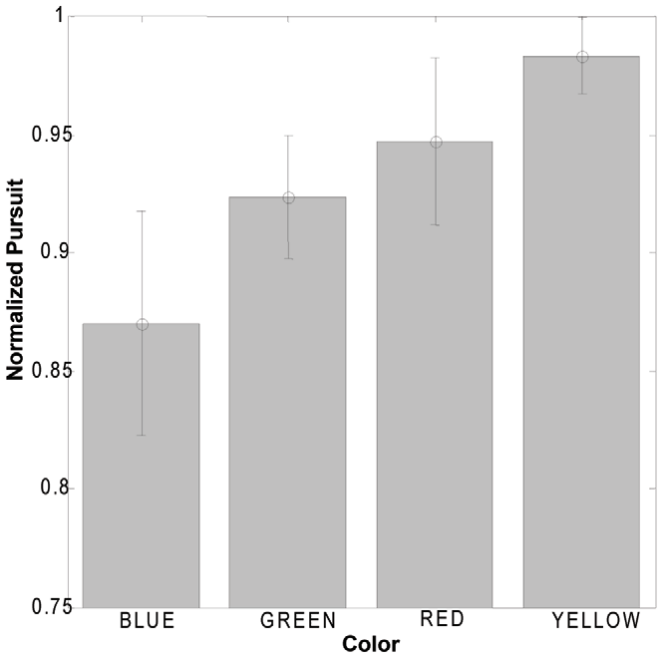
Intrinsic color modulation of pursuit to a single surface. Each subject's data was normalized to the maximum pursuit produced to any color by that subject, to control for between subject variability. The error bars depict SEMs. Subjects exhibited different pursuit levels for different isoluminant colors. A one-way repeated measures analysis of variance showed a significant main effect of color on pursuit. (*F*_(3,12)_ = 4.83, p = 0.0198). This result indicates that colors modulate motion processing which drives the pursuit system.

The results for Experiment 1 revealed that smooth pursuit was modulated by the color of the surface pursued (Figure 2). Even though the colors were equiluminant with each other, changing the color of the surface while maintaining the same velocity and luminance resulted in changes in pursuit velocity. This result indicates that color modulates motion processing which drives the pursuit system. This may have differed from the prior negative result [12] that used a single moving bar and thus local motion processing, whereas our stimulus required global integration of the dot field motion. Global motion integration may involve binding irrelevant features, such as color, in addition to the motion of the individual dots. A key factor is that the stimuli in both of these experiments were also defined by a strong luminance contrast compared to other studies that used isoluminant colored gratings, with no luminance contrast to show weak motion processing of color contrast-defined stimuli [5], [6]. Furthermore, color contrast-defined motion has been shown to have little to no effect when the grating is also luminance-defined [10]. In contrast to this, our results show color modulation of motion processing of luminance contrast defined targets, the more natural occurrence.

**Figure 3:**
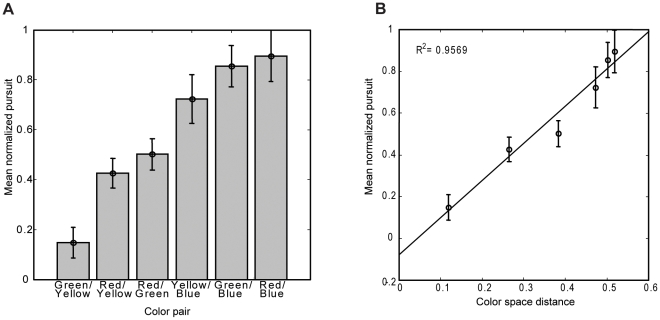
Preferential selection between superimposed surfaces due to color differences. A. Open circles represent mean normalized pursuit (bars: SEM) for each distance between the two colors of each of the six color pairs tested. For each color pair, there was a dominant, selected color. The pattern of results suggests a color hierarchy for selection going from blue (weakest) to yellow to green to red (strongest). B. Pursuit increases as the color space distance for the color pair increases (order of pairs same as in A). A regression analysis revealed a significant and strong relationship between the distance in color space of the two surfaces and pursuit (*R^2^* = 0.9428, *F* = 65.96, *p* = 0.0013). The equation for the regression line is: y = 1.78(x) – 0.079.

Next we investigated whether the effects of color were specific to motion processing or if color differences generalized to stimulus-driven salience and thus would affect automatic target selection. We used the same paradigm except that the aperture consisted of two superimposed colored surfaces moving in opposite directions. We lowered the contrast of the individual dots as we increased the number of dots in the aperture by adding the second surface. Without a task demand to pursue, subjects should select neither of the two surfaces equal in luminance and speed [17]. However, subjects did pursue one of the two superimposed surfaces. These results showed that color differences alone drove target selection. Subjects showed a preference for pursuing red over the other colors, a preference for green over yellow and blue, and a preference for yellow over blue (Figure 3A).

We further investigated the nature of this automatic color-based salience by computing the distance in CIExyY color space for each pair of surfaces (Figure 3B). A regression analysis revealed a significant and strong relationship between the distance in color space of the two surfaces and pursuit (*R^2^* = 0.9428, *F* = 65.96, p = 0.0013). As the color space distance of the two competing superimposed surfaces increased, the amount of pursuit elicited by the selected surface increased as well. Thus there is a color hierarchy for stimulus-driven selection and the strength of selection is dependent upon the distance in color space between the more salient and less salient colors. Isoluminant color, which was irrelevant to the saccade task, drove target selection.

## Discussion

We have found that when all other factors are equal color drives target selection. As color was an irrelevant feature of the surfaces, and there was no task demand to select or pursue either surface, selection by color occurred automatically. The distance between the 2 colors in color space determined the strength of selection. Which surface was selected followed a color hierarchy: red (strongest) to green to yellow to blue (weakest). The red>green>blue hierarchy may arise from the correspondingly ordered cone proportions in the retina [18]. Alternatively, the hierarchy may be due to contextual salience either evolutionary or experiential in nature: red is the color of blood, poisonous berries and stop signals, whereas blue is the color of the sky and thus background in figure-ground segmentation.

Previous studies have shown that motion processing area MT does receive some chromatic input [7]–[10]. However, these color responses have been much weaker than the responses to luminance-defined motion [7]–[10], and color had little to no effect when the stimulus was also defined by luminance [10]. Yet, it has been demonstrated that color differences can be used to filter out distractors from targets in a motion discrimination task [13], [14]. In the latter case, color filtering was part of the task demand for top-down suppression of the distractors. In our paradigm, there were no task demands to select or pursue a given colored surface or even to discriminate motion speed or direction. Aside from color, the surfaces were identical, thus the selection of a surface to pursue and the modulations seen in pursuit speed are not top-down but solely stimulus driven, i.e. a result of the color of the surface.

Our results are consistent with previous results suggesting that basic features, such as binocular disparity and amodal completion, are first bound together into a surface representation which then feeds into motion processing[19], [20]. Our results now suggest a similar mechanism for color selectivity, wherein color and motion are bound at or before the level of areas MT and MST which drive the pursuit system. Pursuit speed was dependent on the distance in color space and such a computation is not associated with motion processing. It is therefore likely that a color sensitive area determines the strength of selection between the 2 surfaces. Binding color into a surface representation that is then fed into the motion processing system would allow for the color hierarchy to bias both target selection and pursuit speed.

High contrast regions automatically capture attention and are included in salience models[21]. Yet we do not live in a black and white world, but one full of color. Current attention and salience models[21], [22] can be extended to include a stimulus-driven color salience module. This color hierarchy for automatic selection impacts a wide range of fields, such as advertising wherein capturing attention is paramount, interface design for efficient data visualization, or even sporting events. For example, a recent study [23] has shown that the color of taekwondo uniforms affected judges' scoring. They took video clips of matches of red and blue uniformed competitors, and digitally altered them so that the uniform colors were reversed. They found that when the competitors had red uniforms, they scored 13% more points than when their uniforms were blue. Our study provides a possible explanation: when red and blue movements occur simultaneously, the red movement is selected over the blue and pursued at a faster speed due to distance between red and blue in color space. Thus when both strikes occur simultaneously, the judges see the red strike first making uniform color the deciding factor. In another example, in gymnastics and in figure skating, the color of the outfit worn would affect the judges' speed of pursuit as they watch the routine which could affect the scoring. This has implications for many sporting events including the Olympics.

## Materials and Methods

Subjects (2 male and 3 female, ages 21–25, 4 naïve) with normal or corrected-to-normal vision and without color blindness (Ishihara Color Plates) participated in the experiments. All participants provided written and informed consent and the research was approved by York University's Human Participants Review Committee. Each participant was fitted with an infrared eyetracker (Eyelink II, SR Research, 500 Hz) to measure eye movements and a bite-bar, for stability, and was placed 57 cm from a 21″ CRT monitor (ViewSonic G225f, 1280x1024, 120 Hz). For every color used, CIExyY color space coordinates were measured with a photometer (SpectraScan PR 655, Optikon Corp). Movies of surfaces comprised of moving coherent dot fields (dot size: 0.04°; dot density: 1.54 dots/deg^2^) were created in Matlab (version R2007a, The Mathworks Corp.) and experimental control was maintained via Presentation (Version 11.0, Neurobehavioral Systems). As shown in Figure 1, when a subject fixated on a central cross (0.5°), a 5° aperture containing a single colored surfaces appeared centered at 7° eccentricity in the lower left or right quadrants and moved either left or right at 6°/s. After a random period of time (500–1500 ms) the fixation cross disappeared which was the signal to saccade to the aperture. The subject's only task was to saccade to the aperture. However, the surface in the aperture continued to move. Smooth eye movements were measured from 50–200 ms post-saccade, discarding trials with saccades during the analysis window (13% across all subjects). We computed the pursuit speed during this window and then normalized the pursuit speed for each color by the maximum speed across all 4 colors to control for between subject variability.

In Experiment 1, the aperture contained a single surface moving either to the left or right. The surface was one of four isoluminant (13.5 cd/m^2^) colors on a black background: red (x = 0.6271, y = 0.3298), green (x = 0.2953, y = 0.5896), blue (x = 0.1511, y = 0.0674), or yellow (x = 0.4014, y = 0.5060). The design was 4 (color) X 2 (direction: left or right) X 2 (hemifield). Subjects completed 30 trials of each condition. In Experiment 2, the aperture contained two superimposed surfaces moving in opposite directions. The surfaces were comprised of 1 of 6 possible color combinations: red-green, red-blue, red-yellow, green-blue, green-yellow and blue-yellow (isoluminant 4.2 cd/m^2^; red: x = 0.6102, y = 0.3272; green: x = 0.2935, y = 0.5640; blue: x = 0.1512, y = 0.0687; yellow: x = 0.3911, y = 0.4886). The design was 6 (color pairs) X 2 (direction of motion) X 2 (hemifield). Subjects completed 30 trials of each condition.
